# Low CYP24A1 mRNA expression and its role in prognosis of breast cancer

**DOI:** 10.1038/s41598-019-50214-z

**Published:** 2019-09-23

**Authors:** Hongqiao Cai, Yan Jiao, Yanqing Li, Zhaoying Yang, Miao He, Yahui Liu

**Affiliations:** 1 Department of Hepatobiliary and Pancreatic Surgery, The First Hospital of Jilin University, Changchun, Jilin 130021 P.R. China; 2 Department of Pathophysiology, College of Basic Medical Sciences, Jilin University, Changchun, Jilin 130021 P.R. China; 3 Department of Breast Surgery, China-Japan Union Hospital of Jilin University, 126 Xiantai Street, Changchun, 130033 P. R. China; 4 Department of Anesthesia, The Second Hospital of Jilin University, Changchun, 130022 P. R. China

**Keywords:** Prognostic markers, Diagnostic markers

## Abstract

Breast cancer is the most common malignant cancer in women. CYP24A1 expression regulates cellular response to vitamin D, which has antitumor effects against breast cancer. This study aimed to identify the correlation between CYP24A1 mRNA expression and prognosis of breast cancer. This study enrolled 1102 patients, including 1090 females and 12 males, from TCGA-BRCA cohort. The Cancer Genome Atlas database was used to study CYP24A1 mRNA expression in breast cancer, and Chi-squared tests were performed to test the correlation between clinical features and CYP24A1 expression. The prognostic value of CYP24A1 in breast cancer was assessed using Kaplan–Meier curves and Cox analysis. Low CYP24A1 expression was associated with age, molecular subtype, ER, PR, HER2, menopause status, N classification, vital status, overall survial and relapse-free survival. CYP24A1 presented a moderate diagnostic ability in breast cancer. Furthermore, low CYP24A1 expression was correlated with poor prognosis. CYP24A1 was an independent risk factor for breast cancer. CYP24A1 plays an important role in prognosis of breast cancer. CYP24A1 has the potential to be a biomarker, especially in predicting prognosis.

## Introduction

Breast cancer is one of the three most common cancers worldwide and has the highest incidence rate of malignancy in women^1^. For breast cancer, biomarkers are particularly useful in identification, diagnosis and predicting prognosis^2^. Although many biomarkers have been in use, they are limited to certain molecular types of breast cancer, thus prompting searches for new biomarkers to predict prognosis on a larger scale.

Vitamin D, the precursor to the potent steroid hormone, calcitriol, has potential anti-proliferative effects on breast cancers^3,4^. A review conducted by Feldman *et al*. has indicated the increased risk of developing cancer with vitamin D deficiency^3^. However, an agreement has not been reached yet whether high or low vitamin D is associated with breast cancer^4^. The vitamin D receptor is expressed in different types of human breast cancers^5^, and active vitamin D has several antitumor effects^6^. The 24-hydroxylase (CYP24A1) enzyme inactivates 1α,25-dihydroxyvitamin D3 (1,25D3), the physiologically active vitamin D metabolite, which regulates cellular response to vitamin D^7,8^. Considering the high heterogeneity of vitamin D signaling in breast cancer, it is unknown whether vitamin D resistance through VDR methylation or CYP24A1 amplification during tumor progression would emerge for one individual’s breast cancer^9^. Thus, CYP24A1 is thought to play an important role in breast cancer through the vitamin D signaling pathway. Recently, CYP24A1 has been studied in many diseases, and it is identified as a potential biomarker for cancers, including lung adenocarcinoma and colorectal cancer^10,11^.

Herein, we evaluated the correlation between CYP24A1 expression in breast cancer and clinicopathologic features through analysis of data from The Cancer Genome Atlas (TCGA) database. We further assessed the independent prognostic value of CYP24A1 expression for overall and relapse-free survival.

## Results

### Patient features

From TCGA database, we obtained RNA expression data and related clinical information. In total, 1102 patients, including 1090 females and 12 males, with breast cancer were analyzed. Moreover, 589 patients were younger than 60 years old, and 513 patients were older than 60 years old. The background of patients was TCGA-BRCA cohort. The average follow-up time of patients for overall survival and relapse-free survival is 1261.6 days and 1262.8 days respectively, and the number of events was 154. The detailed clinical characteristics of these corresponding patients are shown in Table 1, including molecular subtype, TNM stage, survival status and radiation therapy.

**Table 1:** Demographic and clinical characteristics of TCGA cohort.

Characteristics	Numbers of sample size(%)
Age	
<60	589 (53.45)
>=60	513 (46.55)
Gender	
Female	1090 (98.73)
Male	12 (1.09)
NA	2 (0.18)
Histological type	
Infiltrating Ductal Carcinoma	790 (71.56)
Infiltrating Lobular Carcinoma	204 (18.48)
Other	107 (9.69)
NA	3 (0.27)
Molecular subtype	
Basal	142 (12.86)
Her2	67 (6.07)
LumA	422 (38.22)
LumB	194 (17.57)
Normal	24 (2.17)
NA	255 (23.1)
ER	
Indeterminate	2 (0.18)
Negative	239 (21.65)
Positive	813 (73.64)
NA	50 (4.53)
PR	
Indeterminate	4 (0.36)
Negative	345 (31.25)
Positive	704 (63.77)
NA	51 (4.62)
HER2	
Equivocal	180 (16.3)
Indeterminate	12 (1.09)
Negative	565 (51.18)
Positive	164 (14.86)
NA	183 (16.58)
Menopause status	
Inde	34 (3.08)
Peri	40 (3.62)
Post	706 (63.95)
Pre	231 (20.92)
NA	93 (8.42)
T classification	
T1	281 (25.45)
T2	640 (57.97)
T3	138 (12.5)
T4	40 (3.62)
TX	3 (0.27)
NA	2 (0.18)
N classification	
N0	516 (46.74)
N1	367 (33.24)
N2	120 (10.87)
N3	79 (7.16)
NX	20 (1.81)
NA	2 (0.18)
M classification	
M0	917 (83.06)
M1	22 (1.99)
MX	163 (14.76)
NA	2 (0.18)
Stage	
I	182 (16.49)
II	626 (56.7)
III	252 (22.83)
IV	20 (1.81)
X	14 (1.27)
NA	24 (0.91)
Lymph node status	
NO	28 (2.54)
YES	697 (63.13)
NA	379 (34.33)
Margin status	
Close	31 (2.81)
Negative	922 (83.51)
Positive	79 (7.16)
NA	72 (6.52)
Vital status	
Deceased	155 (14.04)
Living	947 (85.78)
NA	2 (0.18)
Radiation therapy	
NO	445 (40.31)
YES	557 (50.45)
NA	102 (9.24)
Neoadjuvant treatment	
NO	1088 (98.55)
YES	13 (1.18)
NA	3 (0.27)
Targeted molecular therapy	
NO	46 (4.17)
YES	533 (48.28)
NA	525 (47.55)
Sample type	
Metastatic	7 (0.63)
Primary Tumor	1097 (99.37)
Overall survival	
NO	933 (85.83)
YES	154 (14.17)
Recurrence-free survival	
NO	816 (89.47)
YES	96 (10.53)
CYP24A1	
High	647 (58.61)
Low	457 (41.39)

Abbreviation: NA, not available.

Note: Inde, indeterminate menopause (neither Pre or Postmenopausal). Peri, perimenopause (6–12 months since last menstrual period). Post, postmenopause (prior bilateral ovariectomy OR >12 mo since last menstrual period with no prior hysterectomy). Pre, prememopause (<6 months since last menstrual period and no prior bilateral ovariectomy and not on estrogen replacement).

### Low CYP24A1 mRNA expression in breast cancer

**Figure 1:**
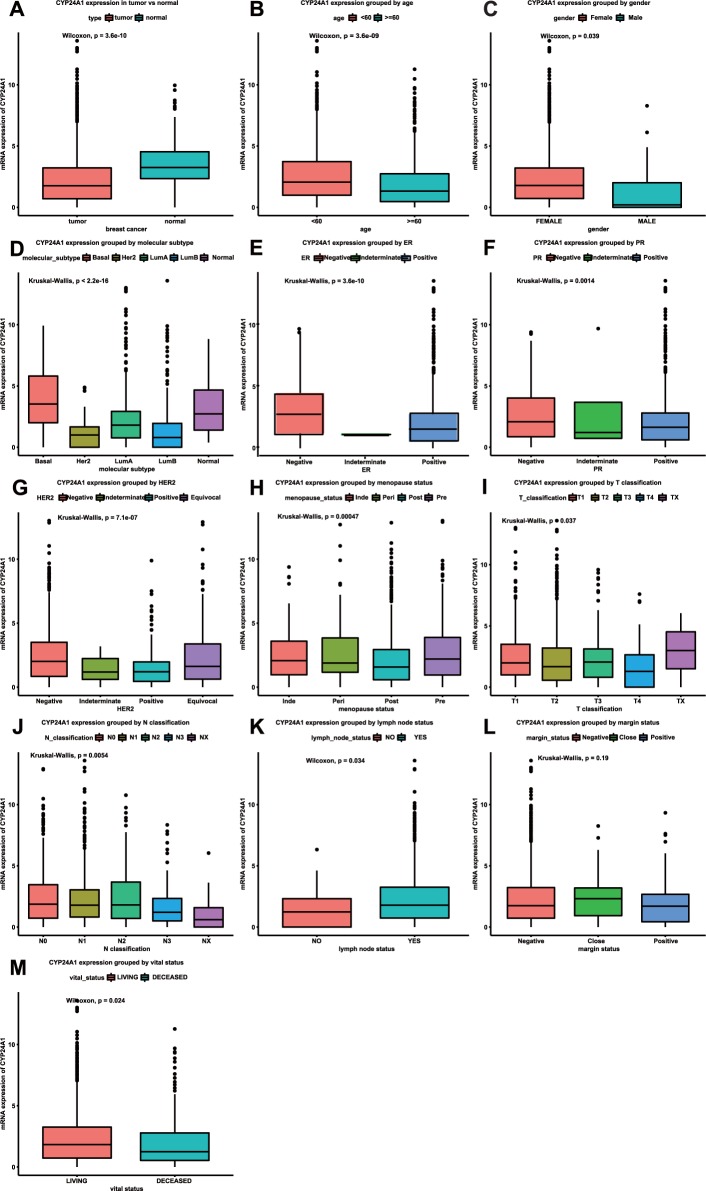
Different CYP24A1 expression levels in the boxplot. CYP24A1 expression in tumor and normal tissue. Expression is grouped by age, gender, molecular subtype, ER, PR, HER2, menopause status, T classification, N classification, lymph node status, margin status and vital status.

As shown in Fig. 1A, the mRNA expression of CYP24A1 in breast tumor tissue was significantly lower than that in breast normal tissue (p = 3.6e-10). Furthermore, different CYP24A1 expression levels were observed in groups based on age, gender, molecular subtype, ER, PR, HER2, menopause status, T classification, N classification, lymph node status, margin status and vital status. Patients who were less than 60 years old had higher CYP24A1 expression levels than patients who were more than 60 years old (Fig. 1B). Female patients had higher CYP24A1 expression levels than male patients (Fig. 1C, p = 0.039), but further studies need to be performed due to the limited number of male patients. With regard to the molecular subtype, only basal breast cancer had higher CYP24A1 expression compared to normal tissue, while Lum A, HER2 and Lum B had lower CYP24A1 expression compared to normal tissue (Fig. 1D). Positive ER, PR and HER2 groups had lower CYP24A1 expression than negative groups (Fig. 1E–G). As shown in Fig. 1H, indemenopausal, perimenopausal and premenopausal groups had similar CYP24A1 expression, while the postmenopausal group had lower CYP24A1 expression compared to the other groups. CYP24A1 mRNA expression levels of different T and N classifications are shown in Fig. 1I,J. Breast cancer with a positive lymph node status had higher CYP24A1 expression than breast cancer with a negative lymph node status (Fig. 1K). Although the p value was greater than 0.05, the group with close margin status had higher expression than the negative and positive groups (Fig. 1L). Deceased patients with breast cancer had lower CYP24A1 expression than living patients with breast cancer (Fig. 1M).

### Capability of CYP24A1 to diagnose breast cancer

**Figure 2:**
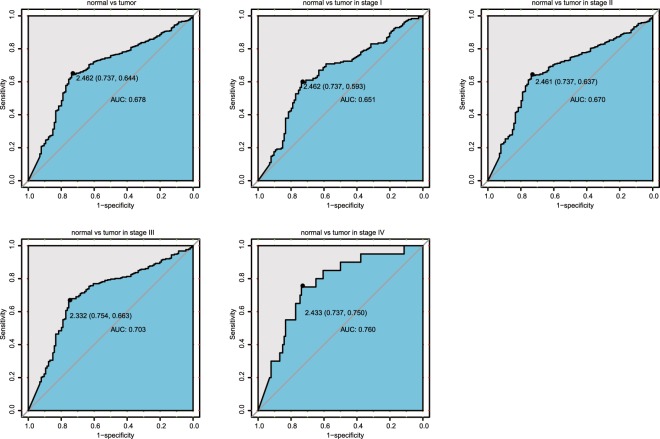
ROC curve of CYP24A1 in breast cancer cohort. Normal and tumor samples in stage 1, stage 2, stage 3 and stage 4.

We used the receiver-operating characteristic (ROC) curve of CYP24A1 to analyze the diagnostic capability of CYP24A1. As shown in Fig. 2, a moderate diagnostic ability in breast cancer was observed with the area under the curve (AUC) of 0.678. We also analyzed the diagnostic capability of CYP24A1 in different stages, and similar results were found with AUC values of 0.651 (stage 1), 0.670 (stage 2), 0.703 (stage 3) and 0.760 (stage 4), showing a progressive increase with higher stages.

### Relationships between clinical characteristics and CYP24A1 expression

We divided the results into two groups based on the medium value for analysis of the relationship between clinical features and CYP24A1 mRNA expression (Table 2). The threshold CYP24A1 level identified from the ROC curve was used to form the low- and high- groups. According to Chi-square tests, low CYP24A1 mRNA expression was highly associated with age, molecular subtype, ER, PR, HER2, menopause status, N classification, vital status, overall survial and relapse-free survival (with P value < 0.01). Moreover, gender (P = 0.0175), histological type (P = 0.034) and neoadjuvant treatment (P = 0.045) were correlated with CYP24A1 expression.

**Table 2:** Correlation between the expression of CYP24A1 and the clinicopathologic characteristics in breast cancer.

Clinical characteristics	Variable	Number	CYP24A1 mRNA		χ^2^	P value
			High n (%)	Low n (%)		
Age	<60	589	394 (60.99)	195 (42.76)	35.6946	**0**.**0005**
	≥60	513	252 (39.01)	261 (57.24)		
Gender	Female	1090	643 (99.54)	447 (98.03)	5.6535	**0**.**0175**
	Male	12	3 (0.46)	9 (1.97)		
Histological type	Infiltrating Ductal Carcinoma	790	448 (69.46)	342 (75)	6.7469	**0**.**034**
	Infiltrating Lobular Carcinoma	204	136 (21.09)	68 (14.91)		
	Other	107	61 (9.46)	46 (10.09)		
Molecular subtype	Basal	142	119 (24.64)	23 (6.28)	99.1391	**0**.**0005**
	Her2	67	25 (5.18)	42 (11.48)		
	LumA	422	255 (52.8)	167 (45.63)		
	LumB	194	66 (13.66)	128 (34.97)		
	Normal	24	18 (3.73)	6 (1.64)		
ER	Indeterminate	2	0 (0)	2 (0.46)	22.9524	**0**.**0005**
	Negative	239	170 (27.6)	69 (15.75)		
	Positive	813	446 (72.4)	367 (83.79)		
PR	Indeterminate	4	2 (0.33)	2 (0.46)	3.8	0.1169
	Negative	345	216 (35.12)	129 (29.45)		
	Positive	704	397 (64.55)	307 (70.09)		
HER2	Equivocal	180	100 (18.59)	80 (20.89)	24.8705	**0**.**0005**
	Indeterminate	12	5 (0.93)	7 (1.83)		
	Negative	565	362 (67.29)	203 (53)		
	Positive	164	71 (13.2)	93 (24.28)		
Menopause status	Inde	34	23 (3.91)	11 (2.6)	15.7947	**0**.**0005**
	Peri	40	27 (4.59)	13 (3.07)		
	Post	706	382 (64.97)	324 (76.6)		
	Pre	231	156 (26.53)	75 (17.73)		
T classification	T1	281	179 (27.71)	102 (22.37)	5.2863	0.2354
	T2	640	363 (56.19)	277 (60.75)		
	T3	138	82 (12.69)	56 (12.28)		
	T4	40	20 (3.1)	20 (4.39)		
	TX	3	2 (0.31)	1 (0.22)		
N classification	N0	516	310 (47.99)	206 (45.18)	13.4385	**0**.**0085**
	N1	367	226 (34.98)	141 (30.92)		
	N2	120	67 (10.37)	53 (11.62)		
	N3	79	37 (5.73)	42 (9.21)		
	NX	20	6 (0.93)	14 (3.07)		
M classification	M0	917	536 (82.97)	381 (83.55)	2.0835	0.3573
	M1	22	10 (1.55)	12 (2.63)		
	MX	163	100 (15.48)	63 (13.82)		
Stage	I	182	117 (18.22)	65 (14.38)	6.4159	0.1599
	II	626	372 (57.94)	254 (56.19)		
	III	252	137 (21.34)	115 (25.44)		
	IV	20	9 (1.4)	11 (2.43)		
	X	14	7 (1.09)	7 (1.55)		
Lymph node status	NO	28	13 (3.02)	15 (5.08)	2.0026	0.1699
	YES	697	417 (96.98)	280 (94.92)		
Margin status	Close	31	20 (3.31)	11 (2.58)	2.6135	0.2599
	Negative	922	545 (90.08)	377 (88.29)		
	Positive	79	40 (6.61)	39 (9.13)		
Vital status	Deceased	155	69 (10.68)	86 (18.86)	14.7927	**0**.**0005**
	Living	947	577 (89.32)	370 (81.14)		
Radiation therapy	NO	445	253 (42.59)	192 (47.06)	1.9543	0.1864
	YES	557	341 (57.41)	216 (52.94)		
Neoadjuvant treatment	NO	1088	641 (99.38)	447 (98.03)	4.1944	**0**.**045**
	YES	13	4 (0.62)	9 (1.97)		
Targeted molecular therapy	NO	46	24 (7.02)	22 (9.28)	0.9821	0.3538
	YES	533	318 (92.98)	215 (90.72)		
Sample type	Metastatic	7	4 (0.62)	3 (0.66)	0.0062	1
	Primary Tumor	1097	643 (99.38)	454 (99.34)		
Overall survival	NO	933	568 (89.31)	365 (80.93)	15.2275	**0**.**001**
	YES	154	68 (10.69)	86 (19.07)		
Recurrence-free survival	NO	816	509 (92.21)	307 (85.28)	11.1183	**0**.**0005**
	YES	96	43 (7.79)	53 (14.72)		

### CYP24A1 mRNA expression is correlated with overall survival

**Figure 3:**
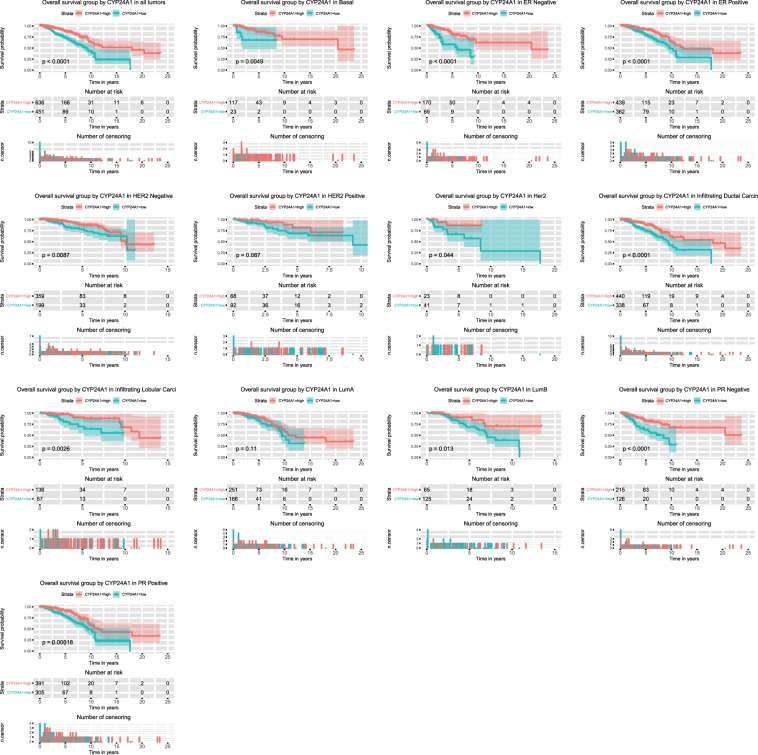
Overall survival analysis of CYP24A1 expression. Kaplan–Meier curves produced overall survival analysis and subgroup analysis of basal, HER2, Lum A, Lum B, HER2-negative tumors, HER2-positive tumors, ER-negative, ER-positive tumors, PR-negative tumors, PR-positive tumors, infiltrating ductal carcinoma and infiltrating lobular carcinoma. The threshold CYP24A1 level identified from the ROC curve was used to form the low- and high- groups.

As shown in Fig. 3, the Kaplan–Meier survival curve with the log rank test revealed the relationship between CYP24A1 mRNA expression and overall survival of patients. Low CYP24A1 expression was significantly associated with poor overall survival (P < 0.0001). The subgroup analysis showed that low CYP24A1 expression indicated a poor overall survival of patients with basal (P = 0.0049), HER2 (P = 0.044), Lum A (P = 0.11) and Lum B (P = 0.013) breast cancer. Additionally, poor overall survival was associated with HER2-negative tumors, HER2-positive tumors, ER-negative tumors, ER-positive tumors, PR-negative tumors, PR-positive tumors, infiltrating ductal carcinoma and infiltrating lobular carcinoma. Univariate Cox analysis identified critical variables, including age, HER2, stage, margin status and CYP24A1. The subsequent multivariate analysis (with 1087 patients actually included) validated that age, clinical stage and CYP24A1 expression were independent prognostic factors for overall survival of patients with breast cancer (Table 3).

**Table 3:** Summary of univariate and multivariate Cox regression analyses of overall survival duration.

Parameters	Univariate analysis			Multivariate analysis		
	Hazard ratio	95% CI	P value	Hazard ratio	95% CI	P value
Age	1.91	1.39–2.63	**0**.**000**	1.95	1.21–3.14	**0**.**006**
Histological type	0.93	0.74–1.17	0.543			
Molecular subtype	1.01	0.88–1.16	0.901			
ER	0.85	0.71–1.02	0.074			
PR	0.87	0.73–1.03	0.096			
HER2	1.29	1.05–1.57	**0**.**013**	1.11	0.89–1.38	0.372
Menopause status	1.16	0.94–1.43	0.165			
Stage	1.64	1.4–1.91	**0**.**000**	2.16	1.64–2.85	**0**.**000**
Lymph node status	1.1	0.93–1.3	0.274			
Margin status	1.42	1.11–1.81	**0**.**005**	0.97	0.69–1.36	0.858
CYP24A1	2.4	1.73–3.31	**0**.**000**	2.01	1.25–3.25	**0**.**004**

### CYP24A1 mRNA expression is associated with relapse-free survival

**Figure 4:**
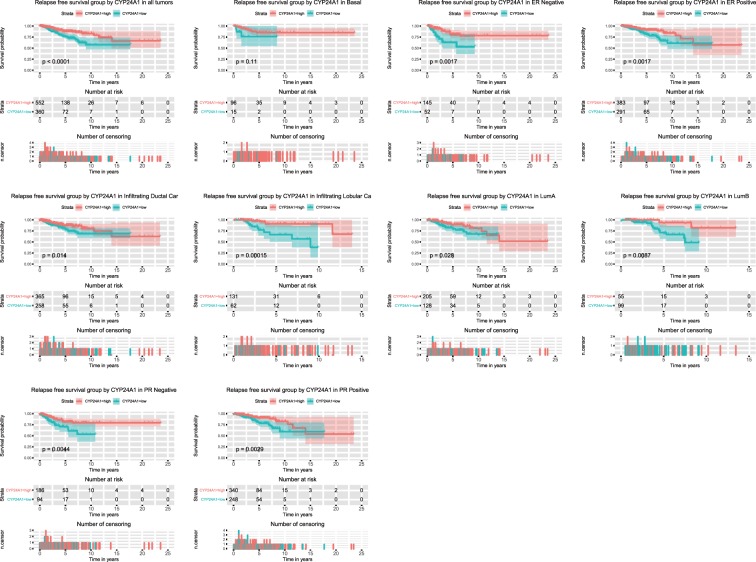
Relapse-free survival analysis of CYP24A1 expression. Kaplan–Meier curves produced relapse-free survival analysis and subgroup analysis of basal tumors, Lum A tumors, Lum B tumors, ER-negative tumors, ER-positive tumors, PR-negative tumors, PR-positive tumors, infiltrating ductal carcinoma and infiltrating lobular carcinoma. The threshold CYP24A1 level identified from the ROC curve was used to form the low- and high- groups.

The Kaplan–Meier survival curve was used for evaluating the relationship between CYP24A1 expression and relapse-free survival (Fig. 4). Similar to the consequences above, low CYP24A1 expression showed a close association with basal tumors, Lum A tumors, Lum B tumors, ER-negative tumors, ER-positive tumors, PR-negative tumors, PR-positive tumors, infiltrating ductal carcinoma and infiltrating lobular carcinoma. Low CYP24A1 expression presented remarkable prognostic value (P < 0.0001). Moreover, univariate Cox analysis was used to select the key prognostic factors (ER, PR, stage, margin status, and CYP24A1), and multivariable analysis was used to adjust the interaction between factors. Furthermore, given that proliferation is a strong prognostic component in ER-positive breast cancer, the correlation between CYP24A1 expression and KI67 (gene MKI67) has been studied. The result showed they are strongly correlated (R^2^ = 0.00219, Fig. S1). CYP24A1 expression was an independent prognostic factor for patients with breast cancer as confirmed by univariate and multivariate Cox analyses (Table 4, with 912 patients actually included in the multivariable Cox analyses).

**Table 4:** Summary of univariate and multivariate Cox regression analyses of relapse-free survival duration.

Parameters	Univariate analysis			Multivariate analysis		
	Hazard ratio	95% CI	P value	Hazard ratio	95% CI	P value
Age	1.45	0.97–2.16	0.072			
Histological type	0.86	0.65–1.14	0.29			
Molecular subtype	0.99	0.82–1.2	0.945			
ER	0.78	0.63–0.97	**0**.**026**	0.74	0.54–1.03	0.075
PR	0.78	0.64–0.96	**0**.**019**	0.87	0.64–1.17	0.345
HER2	0.93	0.7–1.22	0.596			
Menopause status	0.95	0.74–1.22	0.713			
Stage	1.71	1.4–2.08	**0**.**000**	1.64	1.31–2.05	**0**.**000**
Lymph node status	0.86	0.7–1.06	0.159			
Margin status	1.59	1.23–2.06	**0**.**000**	1.43	1.09–1.88	**0**.**009**
CYP24A1	2.22	1.48–3.33	**0**.**000**	2.61	1.68–4.05	**0**.**000**

## Discussion

Our group has recently been studying biomarkers for prognosis of cancers^12–18^. The present study focused on CYP24A1 mRNA expression and demonstrated the important role of CYP24A1 in breast cancer. Low CYP24A1 expression was associated with age, ER, menopause status, TNM classification, stage, margin status, vital status and radiation therapy. In addition, CYP24A1 expression was an independent prognostic factor of breast cancer, making it a promising biomarker with great potential in the near future. However, in contrast with a previously finding that high CYP24A1 expression is upregulated in tumorous breast tissue^6^, we presented a newfound correlation between low expression of CYP24A1 and poor prognosis. The difference may be due to the different ethnicities of people as the tumor samples in the previously reported experiments were collected from the Imam Khomeini Hospital in Iran^6^. Moreover, the sample sizes may have also contributed to the difference (30 vs. 1102 in our study). Although one experiment has suggested that high CYP24A1 expression promotes breast cancer growth^7^, we believe our results and take *in vivo* and *in vitro* discrepancies into consideration.

Analysis of malignant and benign breast tumors obtained from patients after surgery has demonstrated CYP24A1 splicing in breast cancer, and the expression of CYP24A1 protein is significantly reduced in cancerous tissue compared to benign tissue^19^. Our result was consistent with this finding and may be attributed to CYP24A1 splicing because different splicing variants would lead to dysfunction of enzymes, in which enzymes only bind substrates but lack catalytic ability, therefore resulting in abnormal vitamin D levels^19,20^. Low CYP24A1 expression indicates that less CYP24A1 enzyme is produced, leading to more active vitamin D. As two previous studies have disagreed with Yao *et al*., who reported that serum level of vitamin D is associated with lower risk of breast cancer morbidity and mortality, it remains disputable whether vitamin D is good or bad for breast cancer survival^21,22^. The expression of vitamin D receptor was diminished in malignant breast cancer and shown to correlate with a longer relapse-free survival^23,24^. Active vitamin D form (1,25D3) could induce the expression of CYP24A1 through functional vitamin D receptor^25^. However, breast cancer cells may reduce the expression of vitamin D receptor to resist the anti-proliferative effects by vitamin D receptor-mediated vitamin D control^23^. With fewer vitamin D receptors, the inducible expression of CYP24A1 could be limited as well. Survivin suppresses vitamin D, which inhibits cancer cell proliferation, indicating that survivin is an important molecule for the viability of myocytes. Vitamin D inhibits the growth of breast cancer cells. However, considering that breast cancer patients have increased risk for cardiovascular diseases, vitamin D may adversely affect outcomes during the acute phase of cardiovascular conditions, further leading to death caused by noncancer^21^. Because noncancer causes of death are higher than cancer causes of death in breast cancer^26^, we not only focused on the inhibition effect of vitamin D on breast cancer cells but also considered the influence of vitamin D on cardiovascular and other systems as it is a dilemma to obtain a conclusion that high level of vitamin D benefits patients with breast cancer. This point of view was further supported by a newly published article in The New England Journal of Medicine (Manson *et al*.), which demonstrated that supplementation with vitamin D does not result in a lower incidence of invasive cancer or cardiovascular events compared to placebo^27^ with a hazard ratio of 1.02 and 95% CI of 0.79 to 1.31 for breast cancer, indicating no significant difference. Our results were similar to those of that clinical trial as increases in serum vitamin D by intrinsic regulation or extrinsic supplementation may not lower the risk but may be associated with poor prognosis. Similar to results found in other cancers, including lung adenocarcinoma and colorectal cancer^10,11,28^, CYP24A1 may be a promising biomarker in breast cancer. Nevertheless, a consensus has not been reached yet on whether upregulation or downregulation of CYP24A1 leads to poor prognosis when considering the inconclusive function of high vitamin D. Many studies investigated the prognostic role of KI67 in breast cancer and found an increasing value with more evidence^29^. In prognosis, a KI67 level above 10–14% has been suggested to define a group with high risk^29^. Proliferation is a strong prognostic component in ER-positive breast cancer and the strong correlation between CYP24A1 expression and KI67 could possibly further suggest the prognostic value of CYP24A1.

To the best of our knowledge, this is the first study to investigate the correlation between CYP24A1 mRNA expression and prognosis of breast cancer using meta-analysis on a relatively extensive scale. The present study sheds light on the important role of CYP24A1 in breast cancer. However, based on the complexity of the role of vitamin D in breast cancer, the specific function of CYP24A1 needs to be further elucidated by clinical trials in the future.

## Materials and Methods

### Data collection from TCGA database

The RNA expression data was downloaded from the Cancer Genome Atlas (https://cancergenome.nih.gov/) and was shown in RSEM normalized count transformed through calculation using log2(x + 1). The clinicopathological details and related information of breast cancer patients were also collected. This study enrolled 1102 patients, including 1090 females and 12 males, with 589 patients younger than 60 years old. The average follow-up time of patients for overall survival and relapse-free survival is 1261.6 days and 1262.8 days respectively, and the number of events was 154.

### Statistical analysis

For discrete variables, we utilized boxplots to measure the differences of expression by ggplot2 package in R. ROC analysis was performed using R package pROC and Cox regression was performed using R package Survival. SPSS software (Version 19.0) was used to investigate the correlation between CYP24A1 expression and clinical characteristics of breast cancer using Chi-square tests. To compare the overall survival in both groups (high vs. low), Kaplan–Meier curves were used, and P values were calculated. Univariate Cox analysis was performed for selection of related variables. The procedure was repeated for relapse-free survival analysis.

## Supplementary information

Supporting information

## Data Availability

All data is available.
