# Supplementary material for: Low CYP24A1 mRNA expression and its role in prognosis of breast cancer
Source: Sci Rep. 2019 Sep 23;9:13714. doi: 10.1038/s41598-019-50214-z (PMC6757028; doi:10.1038/s41598-019-50214-z)
Supplement: Supplementary file 1 — Supporting information [file 41598_2019_50214_MOESM1_ESM.docx]

Supporting information

# Low CYP24A1 mRNA expression and its role in prognosis of breast cancer

**Authors：**Hongqiao Cai^1^, Yan Jiao^1^, Yanqing Li^2^, Zhaoying Yang^3*^, Miao He^4^, Yahui Liu^1^*

**Affilations：**1 Department of Hepatobiliary and Pancreatic Surgery, The First Hospital of Jilin University, Changchun, Jilin 130021, P.R. China.

2 Department of Pathophysiology, College of Basic Medical Sciences, Jilin University, Changchun, Jilin 130021, P.R. China.

3 Department of Breast Surgery, China-Japan Union Hospital of Jilin University, 126 Xiantai Street, Changchun 130033, P. R. China.

4 Department of Anesthesia, The Second Hospital of Jilin University, Changchun 130022, P. R. China.

**Co-corresponding author:** Zhaoying Yang and Yahui Liu (*)

Email address: Zhaoying Yang: [zhaoyingyang@163.com](mailto:zhaoyingyang@163.com);

Yahui Liu: [liuyahui2008@yeah.net](mailto:liuyahui2008@yeah.net)


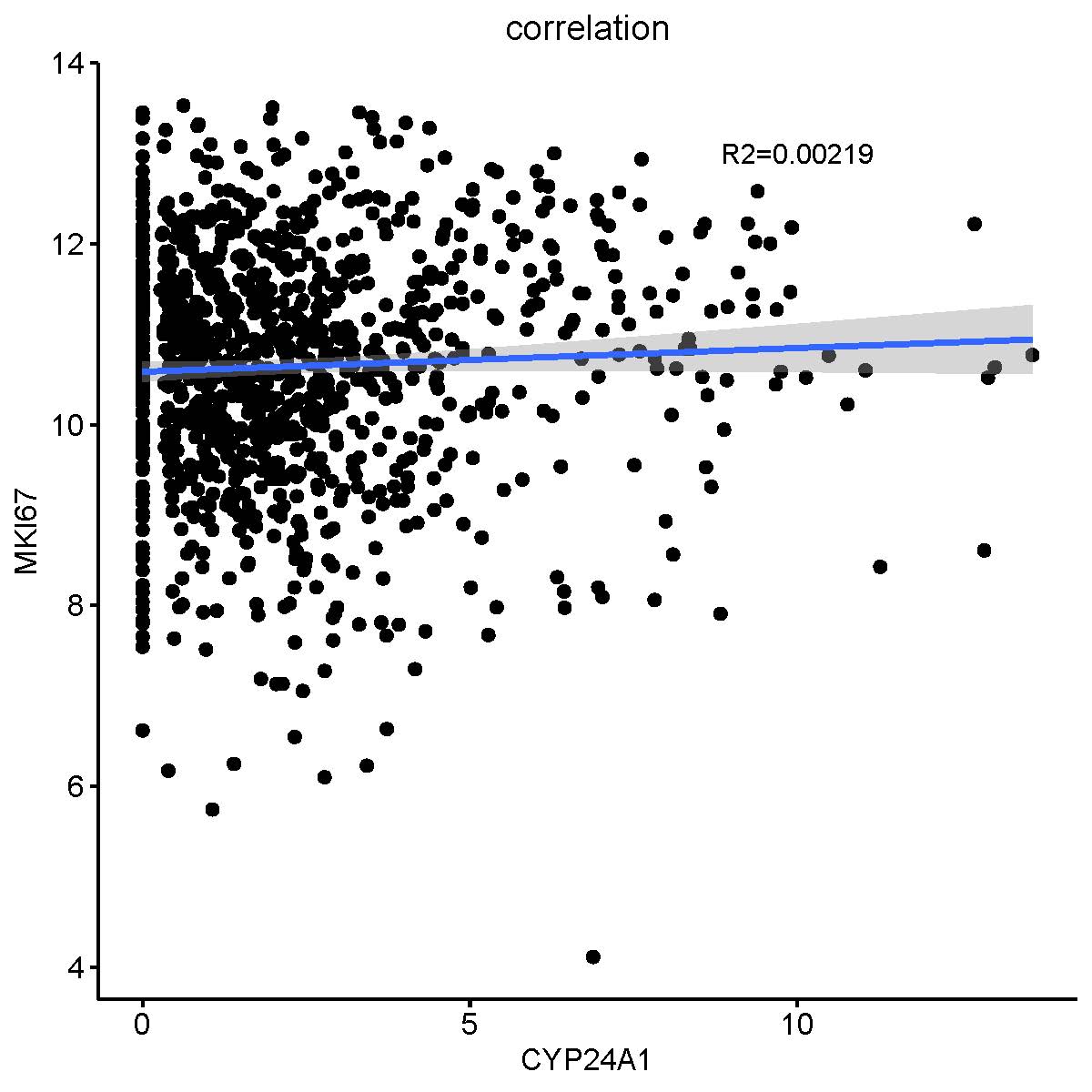
Figure S1. The correlation between CYP24A1 expression and KI67 (gene MKI67).
